# Editorial: Rejuvenation of aging adult stem cells to improve their regenerative potential

**DOI:** 10.3389/fcell.2023.1232970

**Published:** 2023-06-27

**Authors:** Anuradha Vaidya

**Affiliations:** Symbiosis School of Biological Sciences, Symbiosis International (Deemed University), Pune, India

**Keywords:** stem cell niche micro-environment, mesenchymal stromal cells, hematopoietic stem cells, aging, rejuvenation, regeneration

## Abstract

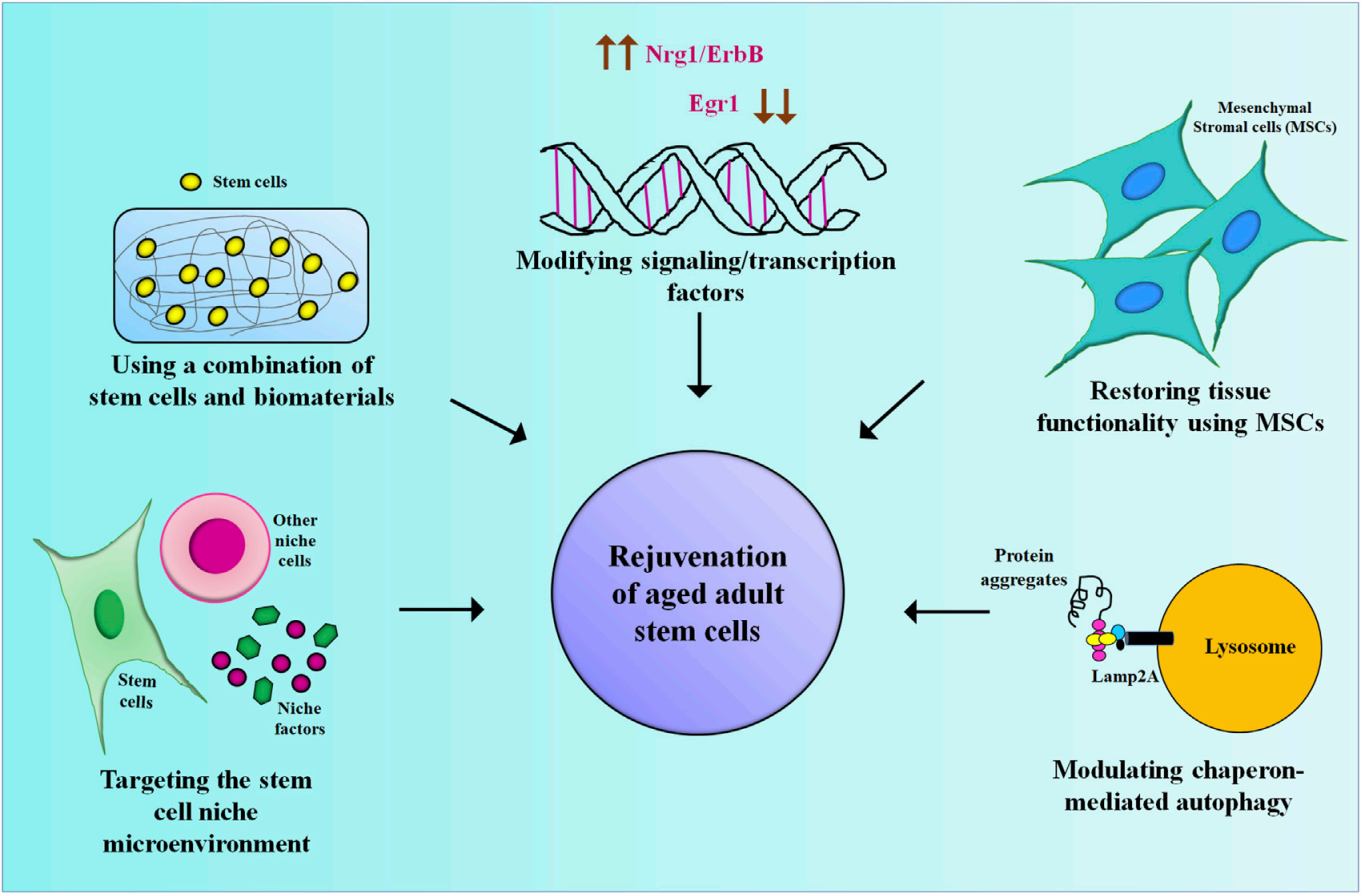

## Introduction

Advances in modern medicine coupled with the advent of smart technologies are allowing people throughout the world to live longer, contributing towards an aged society. At the biological level, aging involves the accumulation of a wide variety of molecular and cellular damage over time. These changes predispose the elderly to irreversible aging-associated conditions such as cancers, neurodegenerative diseases, bone disorders, heart disorders, diabetes, etc (Hou et al., 2019; Finger et al., 2021; Guo et al., 2022).

Adult stem cells (ASCs) present in various tissues play a crucial role in maintaining tissue homeostasis. One of the primary reasons for the functional decline of tissue homeostasis that accompanies aging is the depletion of the stem cell pool in the adult tissue and the simultaneous changes in the stem cell niche and the systemic microenvironment. Together these changes impair the regenerative capacity of ASCs (Carlson and Conboy, 2007; Brunet et al., 2022). Hence, there is a need to identify, develop and optimize novel strategies that rejuvenate the ASCs to restore organ function, which aligns with the scope of this Research Topic. Six articles that discuss various strategies to promote the rejuvenation of ASCs, thereby improving their regenerative potential are published under this Research Topic which includes 3 reviews, 1 mini-review, and 2 original research articles.

### Targeting the aged stem cell niche micro-environment for therapeutic strategies

The extracellular matrix, signaling pathways, various soluble factors, and biochemical elements present in the microenvironment of ASCs are altered by aging. The interaction of ASCs with such an altered microenvironment leads to impaired stem cell functionality and loss of tissue homeostasis. In this review article, Farahzadi et al. have discussed the various niche-targeting strategies for promoting the rejuvenation of aged stem cells. They start by underscoring the importance of the stem cell niche in regulating the fate and behaviour of the stem cells that impacts tissue homeostasis, aging, and disease onset and progression. Finally, they advocate targeting the stem cell niche for developing future therapeutic applications in stem cell transplantation and regenerative medicine.

### Nrg1/ErbB signaling pathway promotes cartilage regeneration in zebrafish

Irreversible cartilage deterioration and incapacitating joint diseases are commonly associated with aging. Since cartilage has a limited potential to repair itself, promoting the growth of cartilage in patients is a major challenge in regenerative medicine. Using zebrafish as a model system, Sapède et al. have shown that the regeneration of chondrocytes is mediated via the Nrg1/ErbB signaling pathway. Upon knockdown of Nrg1 and inhibition of ErbB3/2 receptors, the chondrocytes lose their regenerative potential suggesting that the Nrg1/ErbB pathway could be explored as an alternative target for enhancing the regenerative potential of cartilage in other species as well.

### Perinatal mesenchymal stromal cells of the human decidua as a therapeutic strategy against stress urinary incontinence

Aging alters the structure of the extracellular matrix in the anterior wall of the pelvic tissues, thereby inducing stress urinary incontinence (SUI). In this original research article, De La Torre et al. demonstrate the regenerative potential of decidua-derived mesenchymal stromal cells (DMSCs) using the rat SUI model. When DMSCs are cocultured *in vitro* with the patient-derived myofibroblasts, there is decreased expression of inflammatory cytokines and senescence-related proteins in the myofibroblasts, suggesting DMSCs as a potential therapeutic against SUI.

### Role of early growth response factor 1 in aging hematopoietic stem cells and leukemia

Age-mediated changes in the hematopoietic stem cells (HSCs) microenvironment alter the interaction between the HSCs and the bone marrow niche, thereby affecting the functionality of HSCs. Kulkarni et al. states that early growth response factor 1 (Egr1) is one of the key transcription factors regulating HSC proliferation and their localization in the bone marrow niche. Downregulation of Egr1 improves the proliferation and differentiation of HSCs whereas overexpression of Egr1 leads to inflammation-mediated aging in HSCs. Dysregulation of Egr1 is however associated with hematological malignancies such as acute myeloid leukemia (AML), acute lymphoblastic leukemia (ALL), and chronic myelogenous leukemia (CML). Targeting EGR1 is an encouraging alternative strategy for the rejuvenation of aged HSCs.

### Role of chaperone-mediated autophagy in ageing biology and rejuvenation of stem cells

Chaperone-mediated autophagy (CMA) has emerged as a key mechanism for improving the regenerative potential of stem cells by regulating epigenetic, transcriptional, and differentiation factors in them. Here Vitale et al. have reported the role of CMA in promoting the self-renewal and differentiation potential of embryonic stem cells, its role in the rejuvenation of aged HSCs, and in the regulation of immunosuppressive and differentiation properties of MSCs. They further discuss the significance of CMA to rejuvenate aged stem cells and their application in regenerative medicine.

### Significance of biomaterials in regenerative medicine

Numerous biomaterials have been explored to improve the ability of stem cells to expand and differentiate into various lineages. Nugud et al. have classified biomaterials based on their source, bioactivity, different biological effects, and their role in improving the rejuvenation and differentiation potential of ASCs. Such an approach would help in developing biological substitutes or functional constructs to fix defects in tissues or organs. This review highlights the significance of using biomaterials for stem cell differentiation strategies, thereby enhancing the field of regenerative medicine.
